# Targeted Delivery of Miconazole Employing LL37 Fragment Mutant Peptide CKR12-Poly (Lactic-Co-Glycolic) Acid Polymeric Micelles

**DOI:** 10.3390/ijms222112056

**Published:** 2021-11-08

**Authors:** Takeshi Mori, Miyako Yoshida, Mai Hazekawa, Daisuke Ishibashi, Yoshiro Hatanaka, Rie Kakehashi, Makoto Nakagawa, Toshihiro Nagao, Miki Yoshii, Honami Kojima, Rio Uno, Takahiro Uchida

**Affiliations:** 1 Department of Clinical Pharmaceutics, Faculty of Pharmaceutical Sciences, Mukogawa Women’s University, 11-68 Koshien 9-Bancho, Nishinomiya City 663-8179, Hyogo, Japan; mw319016@mukogawa-u.ac.jp (T.M.); miyakoy@mukogawa-u.ac.jp (M.Y.); h_kojima@mukogawa-u.ac.jp (H.K.); unor@mukogawa-u.ac.jp (R.U.); 2 Department of Immunological and Molecular Pharmacology, Faculty of Pharmaceutical Science, Fukuoka University, 8-19-1 Nanakuma, Jonan-Ku, Fukuoka City 814-0180, Fukuoka, Japan; mhaze@fukuoka-u.ac.jp (M.H.); dishi@fukuoka-u.ac.jp (D.I.); 3 Osaka Research Institute of Industrial Science and Technology, 1-6-50 Morinomiya, Joto-ku, Osaka City 536-8553, Osaka, Japan; hatanaka@omtri.or.jp (Y.H.); rie@omtri.or.jp (R.K.); nakagawa@omtri.or.jp (M.N.); nagao@omtri.or.jp (T.N.); yoshii@omtri.or.jp (M.Y.)

**Keywords:** antimicrobial peptide, scanning electron microscopy, transmission electron microscopy, micelle, drug delivery, drug targeting

## Abstract

We previously reported that conjugates of antimicrobial peptide fragment analogues and poly (lactic-co-glycolic) acid (PLGA) enhance antimicrobial activity and that the conjugated micelle structure is an effective tool for antimicrobial drug delivery. In recent years, the delivery of antimicrobial peptides to targets for antimicrobial activity has attracted attention. In this study, we targeted *Candida albicans*, a causative organism of catheter-related bloodstream infections, which is refractory to antimicrobial agents and is currently a problem in medical practice. We evaluated the antifungal activity of CKR12 (a mutant fragment of the human cathelicidin peptide, LL-37)-PLGA-miconazole (MCZ) micelles using nanotechnology with MCZ delivery. The prepared CKR12-PLGA-MCZ micelles were characterised by measuring dynamic light scattering, zeta potential, dilution stability, and drug release. CKR12-PLGA-MCZ micelles showed higher antifungal activity than CKR12-PLGA micelles and MCZ solution. Furthermore, scanning and transmission electron microscopy suggested that CKR12-PLGA-MCZ micelles disrupted both cell wall and cell membrane of *C. albicans*. Our results revealed a synergistic effect of antifungal activity using a combination of antimicrobial peptide fragment analogues and MCZ, and that MCZ is a promising tool for the delivery to target microorganisms.

## 1. Introduction

Antimicrobial peptides (AMPs) as host defence peptides show potential as a new therapeutic class of antimicrobials [1]. The rapid increase in resistance to conventional antibiotics worldwide has accelerated efforts to introduce AMPs into clinical practice [2]. As linear peptides have low oral bioavailability because of their short half-life, alpha-helical AMP mutagenesis may be a useful approach for increasing antimicrobial activity [3,4,5]. Previous studies have reported that peptides [6] with linked *n*-alkyl acids, hybrid peptides [7] designed by combining α-helical fragments with core antimicrobial cationic fragments, copolymers based on AMPs [8], combinations [9] of AMP and antimicrobial agents [9,10,11,12], hybrid models of AMP and antimicrobial agents [13], hybrids comprised of AMPs with different mechanisms of action [14], dendrimers [15], and nanocarrier models [16] improve antimicrobial activity. However, in our previous study, AMP variants showed reduced antimicrobial activity against *Candida albicans* [17], which is the most common species causing fungal infections [18,19]. de Oliveira Santos et al. [20] found that nearly half of several fungal species isolated from human samples belonged to the genus *Candida*. Systemic candidiasis caused by *C. albicans* is a known cause of death in patients with catheter-related bloodstream infections and opportunistic fungal infections [21]. As *Candida* species continue to evolve, new approaches are needed to treat candidiasis.

The encapsulation of antibiotics into positively charged cationic nanocarriers, which bind to the negatively charged membrane surface of microorganisms, increases bacterial eradication and bioavailability and provides an excellent delivery system using active target nanoparticles [22]. Furthermore, AMPs facilitate delivery of the desired drug by binding to the nanocarrier surface [23]. We hypothesised that CKR12, a mutant of the antibacterial core peptide, uses a similar mechanism, as the representative human cathelicidin AMP LL-37 regulates the expression of genes encoding proteins with various functions in *C. albicans* [24]. This study was conducted to explore the use of a nanocarrier delivery system of micelles [25]. Miconazole (MCZ), an imidazole derivative with a broad antifungal spectrum, was used as the encapsulated sample. Micelles in which MCZ was encapsulated in the CKR12-PLGA were prepared, and the mean particle size, zeta potential, critical micelle concentration (CMC), and drug loading capacity (LC) were measured. Biological evaluation was conducted using *C. albicans*.

## 2. Results

### 2.1. Characterisation of CKR12-PLGA and CKR12-PLGA-Miconazole (MCZ)-Encapsulated Micelles

**Figure 1:**
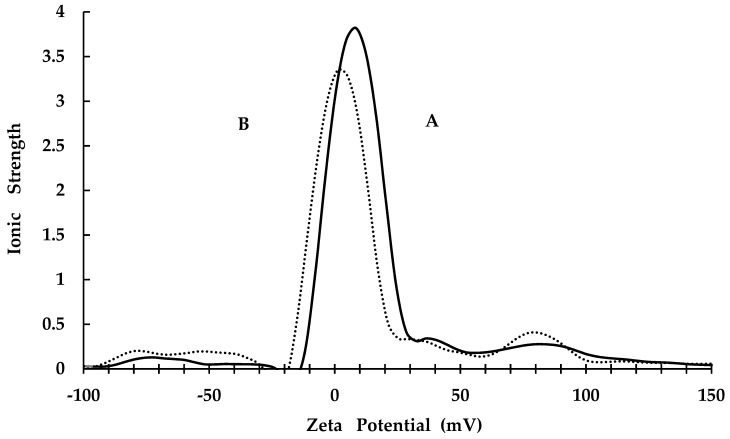
Zeta potential values (CKR12-PLGA is indicated by A, CKR12-PLGA-MCZ is indicated by B).

**Figure 2:**
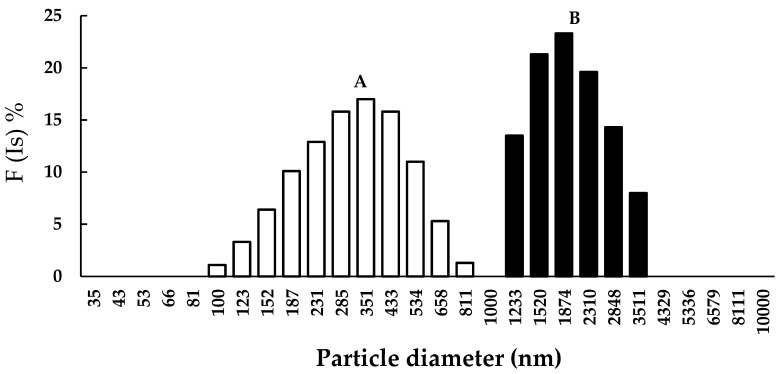
Particle diameter, nm (CKR12-PLGA is indicated by A; CKR12-PLGA-MCZ is indicated by B).

The CKR12-PLGA hybrid block copolymer showed an estimated CMC value of 12 μM based on the absorbance changes reported in our previous study [25]. Therefore, the CKR12-PLGA hybrid block copolymer exhibited the behaviour of an amphiphilic block copolymer in aqueous solution and formed a micelle structure. To confirm successful encapsulation of MCZ, the zeta potential and mean particle size of CKR12-PLGA and CKR12-PLGA-MCZ were measured. The zeta potential of CKR12-PLGA before and after MCZ injection was 8.09 ± 0.07 and 2.86 ± 0.34 (Figure 1), respectively. The average particle size was 278.5 nm before encapsulation and 1946.1 nm after encapsulation (Figure 2). The CMC, zeta potential, and mean particle size suggest that the CKR12-PLGA micelles encapsulated MCZ.

### 2.2. Surface Morphology

**Figure 3:**
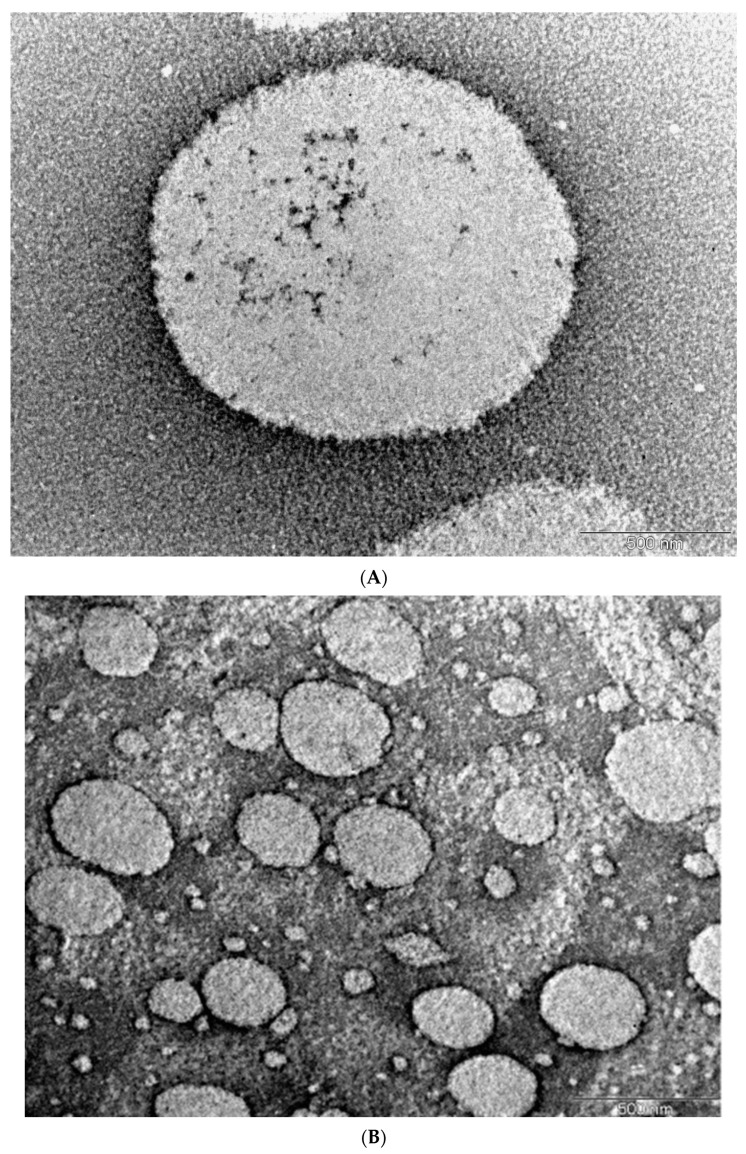
Morphology of CKR12-PLGA-MCZ micelles determined by transmission electron microscopy (**A**) and transmission electron microscopy image of CKR12-PLGA micelles (**B**).

Morphological studies by transmission electron microscopy (TEM) showed that the micelles were spherical with no signs of aggregation and were of diameter approximately 1500 nm, which agreed with the micelle size determined in dynamic light scattering measurements. The image acquired using a blank micelle in Figure 3B shows that the micelle in Figure 3A was enlarged by MCZ inclusion. TEM images of the spherical micelles demonstrate the ability of CKR12-PLGA to assemble into nanoformulations.

### 2.3. Entrapment Efficiency (EE) and LC of MCZ

Because the CMC, zeta potential, and mean particle size results suggested that CKR12-PLGA can form micelles and encapsulate MCZ, we calculated the encapsulation rate and loading dose of MCZ for CKR12-PLGA. In CKR12-PLGA-MCZ micelles, the EE and LC of MCZ were 96.85% ± 6.21% and 23.37% ± 1.63%, respectively.

### 2.4. Dilution Stability Analysis of CKR12-PLGA-MCZ Micelles

**Figure 4:**
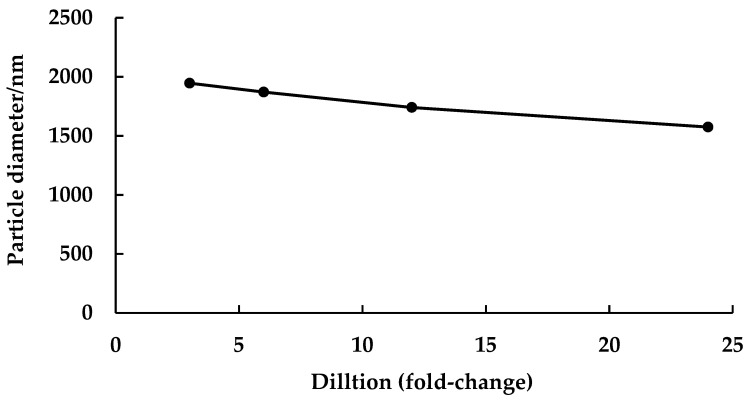
Dilution stability test: size change of CKR12-PLGA-MCZ micelles upon dilution in PBS (pH 7.4).

Dilution stability analysis of CKR12-PLGA-MCZ micelles (MCZ-encapsulated CKR12-PLGA micelles) using physical methods is important for in vitro applications. The dilution stability of CKR12-PLGA-MCZ micelles is shown in Figure 4. The particle diameter at a 3-fold dilution (CKR12-PLGA concentration = 128.5 μM) in phosphate-buffered saline (PBS) (pH 7.4) was 1946.1 nm, and that at a 24-fold dilution (CKR12-PLGA concentration = 16.1 μM) was 1574.4 nm. At both dilutions, CKR12-PLGA-MCZ micelles were stable. However, the particle diameter at a 48-fold dilution (CKR12-PLGA concentration = 8 μM) in PBS (pH 7.4) was unmeasurable. CKR12-PLGA-MCZ micelles were stable until 16.1 μM CKR12-PLGA, which was more than the CMC of CKR12-PLGA, that is, 12.34 μM.

### 2.5. In Vitro Drug Release

**Figure 5:**
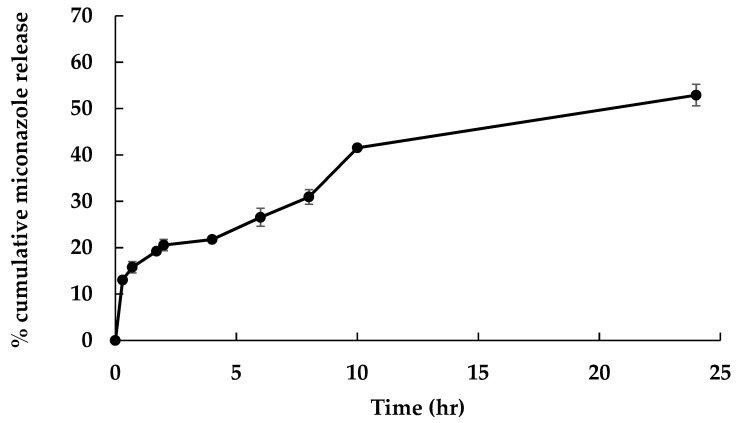
In vitro release profile of MCZ in CKR12-PLGA-MCZ micelle. N = 5, mean ± S.D.

The in vitro release profile of MCZ in CKR12-PLGA-MCZ micelles in PBS at pH 7.4 was measured by high-performance liquid chromatography (HPLC) (Figure 5).

The in vitro release profile of MCZ in CKR12-PLGA-MCZ micelles showed time-dependent drug release at a neutral physiological pH (pH 7.4). The drug release rate of MCZ in CKR12-PLGA-MCZ micelles after 24 h was 52.90% ± 2.32%.

### 2.6. Antifungal Activity

After identifying CKR12-PLGA-MCZ micelles, the antifungal activity of MCZ, CKR12- PLGA, and CKR12-PLGA-MCZ was examined (Table 1).

The MICs show that CKR12-PLGA-MCZ micelles had the highest antifungal activity against *C. albicans* followed by MCZ and CKR12-PLGA. The antifungal activities of CKR12-PLGA and MCZ were similar, and CKR12-PLGA-MCZ was 100-fold more potent than the other two drugs. The effects of PLGA alone (181.00 ± 0.00 μM) were confirmed, revealing minimal antimicrobial activity against *C. albicans*.

### 2.7. Combined Treatment against C. Albicans with CKR12-PLGA and MCZ

The checkerboard method was used to evaluate the activity of a combination of antibiotics tested at clinically relevant concentrations in a series of double dilutions. The combination of assays was designed to include different classes of antimicrobial agents. The data obtained using the checkerboard method were analysed as the fractional inhibitory concentration index (FIC). The FIC was calculated by comparing the MIC value of each drug alone with that of the combination; combinations of antibiotics that decrease the MIC by 4-fold compared with each drug alone were considered synergistic (FIC ≤ 0.5). FICs of 0.5–1.0 were considered to indicate non-synergistic or additive effects. As the FIC values of 1–4 were defined as indifferent and those >4 were antagonistic, the FIC was calculated using the MIC values.

Table 2 shows the FIC index calculated from the MIC of CKR12-PLGA-MCZ micelles. The FIC_A_ is the MIC of CKR12-PLGA itself (0.24 µM), observed when CKR12-PLGA and MCZ were combined, divided by the MIC of CKR12-PLGA alone (24.2 µM) (FIC_A_ = 0.01). The FIC_B_ is the value obtained by dividing the MIC (3.12 µM) of MCZ, which is observed when MCZ are used in combination with CKR12-PLGA, by the MIC (24.03 µM) of MCZ alone (FIC_B_ = 0.13). In the combination of CKR12-PLGA and MCZ, the FIC_A_ = 0.01 and FIC_B_ = 0.13; therefore, the FIC index = FIC_A_ + FIC_B_ = 0.14. From the above results, it was found that the synergistic effect of CKR 12-PLGA and MCZ was obtained because the FIC index was 0.14, which was less than 0.5.

### 2.8. Scanning Electron Microscopy (SEM) and TEM Images

**Figure 6:**
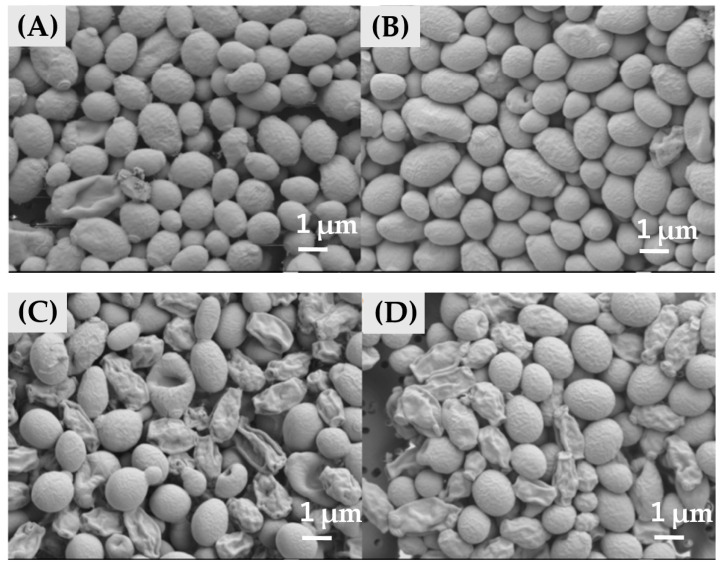
Scanning electron microscopy images of *C. albicans* cells. (**A**) Control; (**B**) in the presence of CKR12-PLGA = 64 µM; (**C**) in the presence of MCZ = 199 µM; (**D**) in the presence of CKR12-PLGA-MCZ = 64 µM.

Next, we examined the effect of CKR12 -PLGA-MCZ micelles on the morphology of *C. albicans*. The results of SEM are shown in Figure 6. In the presence of CKR12-PLGA, the cell surface was slightly coarse and some cells were crushed (Figure 6B). In the presence of MCZ, most cells had a coarse surface and were crushed (Figure 6C). In the presence of CKR12-PLGA-MCZ micelles, the cells had a slightly coarse surface or were crushed (Figure 6D).

**Figure 7:**
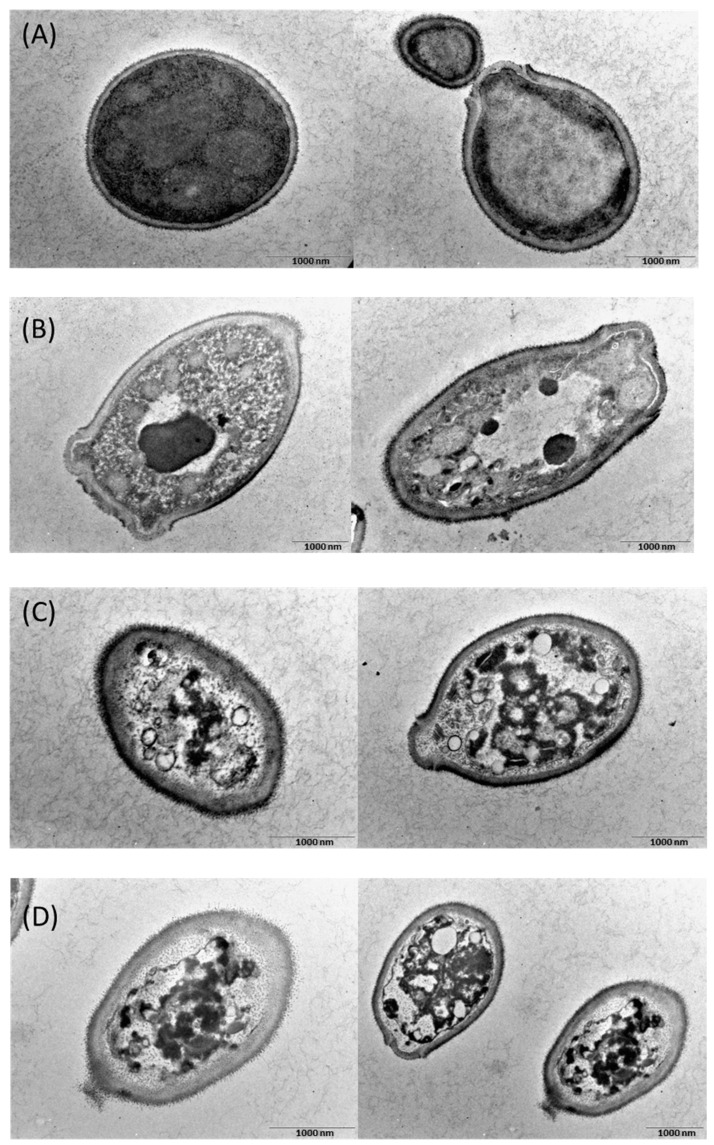
Transmission electron microscopy images of *C. albicans* cells. (**A**) Control; (**B**) in the presence of CKR12-PLGA = 64 µM; (**C**) in the presence of MCZ = 199 µM; (**D**) in the presence of CKR12-PLGA-MCZ micelles = 64 µM.

Figure 7 shows the TEM images of *C. albicans*. The cells treated with CKR12-PLGA, MCZ, or CKR12-PLGA-MCZ micelles were spongy (Figure 7B–D). Particularly, in cells treated with CKR12-PLGA-MCZ micelles, the cell wall and cell membrane were disrupted (Figure 7D). These results indicate that cellular changes are the decisive factors in deformation, which is consistent with the results of electron microscopy.

## 3. Discussion

The significance of the micellarisation of MCZ with CKR12-PLGA is that this formulation can maximise the antifungal activity of MCZ by eliminating the resistance of *C. albicans* to azoles and increasing the intracellular penetration of MCZ through structural and physicochemical effects. CMC, zeta potential, and average particle size measurements and dilution stability and release studies were conducted to evaluate the effect of micelles on MIC enhancement. The SEM results showed that CKR12-PLGA micelles attack the cell surface of *C. albicans*. When solubilising poorly water-soluble substances in amphiphilic surfactants, the relationship between the ratio of the surfactant and poorly water-soluble substance enables micelle formation, although micelle formation is not dependent on the ratio [26]. Therefore, the mixing ratio need not be considered. Furthermore, the position of solubilisation of poorly water-soluble substances in micelles is influenced by the structural features of the substances. Substances with lower molecular weights, polar molecules, and aromatics tend to be solubilised in the palisade layer, whereas substances with larger molecular weights and nonpolar molecules tend to be solubilised in the core [27,28,29]. CKR12-PLGA is an amphiphilic polymer, and the poorly water-soluble MCZ with an aromatic backbone can be solubilised in the palisade layer of the micelle by “permeation”. Based on these results, the formation of spaces between CKR12 on the micelle surface led to a decrease in the zeta potential (8.09 ± 0.07 mV → 2.86 ± 0.34 mV) and an increase in the particle size (278.5 nm → 1946.1 nm).

The zeta potential of the CKR12-PLGA-MCZ micelles was 2.86 ± 0.34 mV. The zeta potential of *C. albicans* is approximately −20 to 35 mV. The size of the microorganisms is also small; additionally, despite its hydrophobic nature, the biodegradable polymer PLGA shows high lipophilicity [30]. Therefore, micelles with a positive surface charge and *C. albicans* with a negative surface charge attract each other through electrostatic interactions. Therefore, micelles tend to bind to the negatively charged *C. albicans*.

The MIC results showed that the CKR12-PLGA conjugate and MCZ have similar antifungal activities because of the synergistic effect of the CKR12-PLGA conjugate and MCZ even at concentrations lower than each MIC, although this effect was dependent on the environment and conditions in which the micelles were used. These results indicate that the CKR12-PLGA-MCZ micelles can be used as a nanocarrier, and that encapsulation of MCZ in a positively charged cationic nanocarrier that can bind to the negatively charged membrane surface of microorganisms can be used to increase microbial eradication and bioavailability. The presence of CKR12 on the micelle surface allows it to easily bind to the nanocarrier membrane surface and exhibit antifungal activity because of its cell membrane permeability. Therefore, the use of positively charged cationic nanocarrier formulation with these two mechanisms of action is a promising approach.

The dilution test showed that the particle diameter of CKR12-PLGA-MCZ micelles was not changed until a dilution of 24-fold (16.1 μM). The concentration of CKR12-PLGA-MCZ was almost the same as the CMC of CKR12-PLGA (12 μM) because in the case of amphiphilic micelles, in which the micelle core is a hydrophobic polymer, incorporation of insoluble substances increases micelle stability [31]. Next, the characterisation of the in vitro release profile of the CKR12 PLGA-MCZ micelles showed that it was a sustained release formulation that could retain MCZ for a long time. As the formulation was designed to ensure that MCZ would not be lost before the micelles reached the target *Candida*, the antifungal activity can be exerted for a long period.

For CKR12-PLGA, even if there are no noticeable changes in the cell surface in SEM, the effects may be equivalent to those of MCZ in TEM; that is, more than half of the observed cells are intracellular sponges. Therefore, even if the effect was not observed on the extracellular surface, it was sufficient on the intracellular surface; CKR12-PLGA-MCZ micelles more clearly showed strain and disruption of the cell surface than CKR12-PLGA. This is consistent with the measured antifungal activity; the cell membrane of *C. albicans* is protected by the cell wall without directly contacting the external environment. By targeting the cell membrane, MCZ must first interact with the cell wall [32]. After penetrating the cell wall and altering the cell membrane, MCZ affects the cell wall structure to make it brittle. Therefore, the combination of CKR12-PLGA micelles with MCZ is expected to increase the intracellular penetration of MCZ because the micelles physically disrupt the cell wall, and thus, a small amount of MCZ can be used to achieve the maximum effect. In conclusion, CKR12-PLGA-MCZ micelles have the potential to deliver drugs to targeted microorganisms. The amount of MCZ used in this study showed excellent antimicrobial activity at concentrations as low as one-eighth of the general antifungal activity concentration. This may be because of physical disruption of the cell wall by AMPs from the outside and chemical disruption by MCZ from the inside. Through this approach, a synergistic effect was achieved.

The CKR12-PLGA-MCZ micelles are a mixture of CKR12-PLGA micelles, CKR12-PLGA conjugate present in micelle disintegration, and MCZ. The interaction of CKR12-PLGA and MCZ under CMC (synergistic effect) produced an ideal mechanism of action, showing stable antifungal activity over time. These findings may be a result of the physical disruption of the cell wall of *C. albicans* by CKR12 [33] and the chemical disruption of the cell membrane by MCZ [29], resulting in enhanced antifungal activity.

In the present study, we aimed to improve antimicrobial activity and targeting by combining CKR12, an AMP fragment analogue with broad-spectrum activity, and PLGA to prepare micelles that encapsulate the therapeutic drug MCZ. CKR12 showed antimicrobial activity and was present on the micelle surface, and MCZ showed sufficient antimicrobial activity even at low concentrations by being continuously released from the micelles. This result highlights the synergistic effect between the antimicrobial activity of MCZ and CKR12 and provides a promising tool for delivering therapeutic agents to target microorganisms.

## 4. Materials and Methods

### 4.1. Materials

The LL-37 fragment (17–29) was purchased from Funakoshi Co., Ltd. (Tokyo, Japan). The mutant peptide (CKRIVKRIVKKWLR) was synthesised by Toray Research Center (Tokyo, Japan). Poly (d, l-lactic-co-glycolic acid) (PLGA7510, MW: 10,000), 3-(2-pyridyldithio) propionylhydrazide, *N*, *N*-dicyclohexylcarbodiimide, and *N*-hydroxysuccinimide were purchased from Wako Pure Chemical Industries (Osaka, Japan). *Candida albicans* (JCM 1542) was purchased from the Japan Collection of Microorganisms in the Riken BioResource Research Center (Ibaragi, Japan).

### 4.2. Methods

#### 4.2.1. Synthesis and Characterisation of CKR12-PLGA Hybrid Block Copolymer

The procedures for synthesis and structural characterisation by Fourier transform-infrared spectroscopy, ^1^H-nuclear magnetic resonance spectroscopy, and haemolytic assays of the CKR12-PLGA hybrid block copolymer have been reported previously [25].

#### 4.2.2. CMC

The CMC is defined as the lowest concentration of a surfactant at which micelles form in water. The CMC was determined from the absorbance of Sudan III at the maximum absorption wavelength (511 nm) via the dye solubilisation method using a spectrophotometer [34]. From the two-line equation obtained by processing the absorbance of the CKR12-PLGA hybrid block copolymer solution (sample concentrations 0, 4.38, 8.76, 17.52, 35.05, and 57.83 μM) via linear fitting, the concentration on the abscissa was defined as the CMC of CKR12-PLGA.

#### 4.2.3. Preparation of MCZ-Loaded Micelles (CKR12-PLGA-MCZ)

CKR12-PLGA-MCZ micelles were prepared using a solubilisation method. Briefly, a solution of MCZ (0.98 mg) in acetonitrile (100 μL) was added to a solution of CKR12-PLGA (500 μL) and sonicated for 1 min. The resulting emulsion was stirred overnight to completely remove acetonitrile at room temperature.

The total amount of MCZ entrapped in CKR12-PLGA-MCZ micelles was determined using an ultrafiltration method [35]. Briefly, the micelle solution was filled into an Amicon Ultra 3 K device (Merck, Kenilworth, NJ, USA) with a molecular weight cut-off of 3 kDa and centrifuged at 14,000× *g* for 5 min at 25 °C. The amount of MCZ encapsulated in CKR12-PLGA was calculated by measuring MCZ dissolved in the filtrate by HPLC [36].

#### 4.2.4. Determination of Particle Size and Zeta Potential

The size of the CKR12-PLGA micelles and CKR12-PLGA-MCZ micelles was measured using a dynamic light scattering instrument (Otsuka Electronics Co., Ltd., Osaka, Japan), and the surface charge was measured using ELSZ-2000 (Otsuka Electronics Co., Ltd., Osaka, Japan). The CKR12-PLGA micelles were dissolved in 1 mM sodium chloride and ultrapure water; the effective hydrodynamic diameter and zeta potential of CKR12-PLGA micelles and CKR12-PLGA-MCZ micelles were measured in triplicate.

#### 4.2.5. Surface Morphology

The surface morphology of CKR12-PLGA-MCZ micelles was investigated by TEM. A drop of suitably diluted micelles was allowed to dry on a copper grid (300 mesh) coated with 3 mM forman (0.5% plastic powder in amyl acetate), and uranyl acetate stain (2% *w*/*v*) was added for 30 s and dried. An accelerating voltage of 100 kV was used to visualise the micelles using a mega view III camera (JEM-1010, Jeol, Tokyo, Japan).

#### 4.2.6. EE and Drug LC

The total amount of MCZ entrapped in CKR12-PLGA-MCZ micelles was determined using an ultrafiltration method [35]. Briefly, the micelle solution was filled into an Amicon Ultra 3 K device (Merck, Kenilworth, NJ, USA) with a molecular weight cut-off of 3 kDa and centrifuged at 14,000× *g* for 5 min at 25 °C. The amount of MCZ encapsulated in CKR12-PLGA was calculated by measuring MCZ dissolved in the filtrate by HPLC [36].

The HPLC methods used for MCZ were based on the Japanese Pharmacopoeia and a previous report [24]. For HPLC, 1 µL of sample was injected into a chromatograph (LC-10AT VP; Shimadzu Corporation, Kyoto, Japan) equipped with a UV detector (SPD-10A VP; Shimadzu Corporation, Kyoto, Japan), an integrator (LC solution; Shimadzu Corporation, Kyoto, Japan), and a reverse-phase column (CAPCELL PAK C18 UG120 S5: 150 mm × 4.6 mm i.d.; Shiseido Co., Ltd., Tokyo, Japan). The column temperature was maintained at 30 °C. The mobile phase composition for MCZ was (A) an aqueous solution of 2.7% (*w*/*v*) tetra-*n*-butyl ammonium hydrogen sulphate and (B) acetonitrile, respectively, and the flow rate was 1.0 mL/min. The run time for MCZ was 10 min. The ultraviolet detection wavelength of MCZ was set to 225 nm.

All experiments were performed in triplicate. The following formulae were used to calculate the EE and LC for CKR-12-PLGA-MCZ micelles [33,37]:

%EE = (weight of MCZ in micelles/weight of MCZ added) × 100,

%LC = (weight of MCZ in micelles/weight of micelles) × 100.

#### 4.2.7. Dilution Stability of CKR12-PLGA-MCZ Micelles

The dilution stability of CKR12-PLGA-conjugated-MCZ micelles was evaluated as previously reported with slight modifications [35]. Briefly, the micelle sample (containing 0.66 mg/mL of CKR12-PLGA-conjugated) was diluted in PBS (pH 7.4) from 3–48-fold at room temperature, and the changes in the particle size were determined using DLS-6000HLC (Otsuka Electronics Co., Ltd., Osaka, Japan). The changes in the surface charge were determined using ELSZ-2000 (Otsuka Electronics Co., Ltd.).

#### 4.2.8. In Vitro Drug Release

The drug release test was performed as previously described with some modifications [23]. Briefly, 0.75 mL of CKR12-PLGA-MCZ micelles (MCZ concentration = 1.60 mM) (donor solutions) was added into dialysis tubes (MWCO 6–8 kDa, Spectrum Laboratories, Inc., San Francisco, CA, USA), and dialysis was performed against 30 mL of PBS of pH 7.4 (receiver solution) at 37 °C. At fixed time intervals, the samples (0.75 mL) were collected from the receiver solutions for analysis and replaced with an equal volume of fresh PBS (pH 7.4) to maintain sink conditions. For the HPLC analysis, 10 µL of the sample solution was injected into a chromatograph (LC-10AT VP; Shimadzu Corporation) equipped with a UV detector (SPD-10 A VP; Shimadzu Corporation), an integrator (LC solution, Shimadzu), and a reversed-phase column (CAPCELL PAK C 18 UG 120 S5: 150 mm × 4.6 mm i.d.; Shiseido Co., Ltd.). The mobile-phase composition of MCZ was (A) 2.7% (*w*/*v*) ammonium hydrogen tetra-*n*-butyl sulphate and (B) aqueous solution of acetonitrile (65:35, %) at a flow rate of 1.0 mL/min. The measurement time of MCZ emitted after each time interval was 7 min. The UV detection wavelength of MCZ was 225 nm. The mobile-phase composition of MCZ was acetonitrile (2.7%, *w*/*v*) and tetra-*n*-butylammonium hydrogen sulphate (35:65, *v*/*v*).

#### 4.2.9. In Vitro Antimicrobial Activity

The antimicrobial activity of MCZ, CKR12-PLGA, and CKR12-PLGA-MCZ micelles was characterised by determining their MICs. The microbial strain *C. albicans* in the exponential growth phase was diluted to 4 × 10^4^ CFU/mL and then to 4 × 10^4^ CFU/mL in YM broth and dispensed into each well of a 96-well microtiter plate. Susceptibility tests were performed using two-fold standard broth microdilution of MCZ, CKR12-PLGA, and CKR12-PLGA-MCZ micelles according to Clinical and Laboratory Standards Institute guidelines [38]. The MICs were measured using the test samples of MCZ (384.49–0.18 µM), CKR12-PLGA (192.82–0.09 µM), and CKR12-PLGA-MCZ (64.27–0.06 µM). Each analysis was performed in triplicate. The lowest concentration (highest dilution) required to observe no growth of *C. albicans* was considered the MIC. The MIC values were determined by visual inspection.

#### 4.2.10. Combined Treatment of *C. albicans* with CKR12-PLGA and MCZ

The effect of the combination of CKR12-PLGA and MCZ on *C. albicans* (JCM 1542) was evaluated by calculating the equivalent amount of each sample of CKR12-PLGA and MCZ from the results of the MIC test using the standard checkerboard method and the FIC index [39].

FIC = FIC_A_ + FIC_B_.

FIC_A_ = (MIC_A_ in combination)/(MIC_A_ alone)

FIC_B_ = (MIC_B_ in combination)/(MIC_B_ alone)

#### 4.2.11. Imaging by SEM and TEM

For microscopic measurements, the microbes were prepared as follows. First, a cell suspension of *C. albicans* (5.5 × 10^8^ CFU/mL) was used. For SEM and TEM imaging, the suspension was incubated with MCZ (199 μM), CKR12-PLGA (64 μM), or CKR12-PLGA-MCZ (64 μM) at 27 °C. To evaluate the morphological changes with the drug, considerably higher drug concentrations than the MICs were applied for SEM and TEM imaging. After 24 h, for SEM, 50 μL of the mixed solution was collected and the cells were washed five times in PBS, five times in 2.5% glutaraldehyde, five times in PBS, fixed with osmic acid, washed five times in purified water, dehydrated with ethanol, replaced in *t*-butyl alcohol, coated with gold, and observed under a scanning electron microscope (JSM-7800; Jeol, Tokyo, Japan). For TEM, 150 μL of the recovered mixed solution was mixed with low-melting-point agar and fixed with glutaraldehyde. The prepared pellets were imaged by TEM (JEM-2100; Jeol, Tokyo, Japan).

## 5. Conclusions

The use of AMPs on the surface of CKR12-PLGA-MCZ micelles is a novel approach for microbial targeting and maximising the performance of MCZ. This micellar approach has the potential to change the concept of dosage in existing drug therapies. The results revealed that the MCZ concentration, which normally does not show antifungal activity, had the greatest therapeutic effect. These findings will greatly contribute to future drug therapies.

## Figures and Tables

**Table 1:** Minimum inhibitory concentration (MIC) of MCZ, CKR12-PLGA, and CKR12 PLGA-MCZ against *C. albicans*. The MIC (μM) was determined as the average values of three experiments.

Organism	MIC Value (μM)			
	CKR12-PLGA	CKR12-PLGA-MCZ	MCZ	PLGA
*C. albicans* (JCM 1542)	24.25 ± 0.00	0.24 ± 0.00 Encapsulate amount (MCZ 3.12)	24.03 ± 0.00	181.00 ± 0.00

**Table 2:** Combined activity of CKR12-PLGA and MCZ.

Drug A	Drug B	FIC_A_	FIC_B_	FIC	Action
CKR12-PLGA	MCZ	0.01	0.13	0.14	Synergism
